# Predictive and Incremental Validity of Parental Representations During Pregnancy on Child Attachment

**DOI:** 10.3389/fpubh.2020.439449

**Published:** 2020-12-02

**Authors:** Renata Tambelli, Cristina Trentini, Francesco Dentale

**Affiliations:** Department of Dynamic and Clinical Psychology, “Sapienza” University of Rome, Rome, Italy

**Keywords:** pregnancy, parental pre-natal representations, parent–child interactions, emotional availability, child attachment

## Abstract

Parental pre-natal representations predict the interactive patterns that parents will put in place after childbirth. Early interactions defined by high parental emotional availability (EA) influence the development of security in children. To date, research on the predictive role of parental pre-natal representations on child attachment is still poor. Moreover, investigations on pre-natal representations have mainly focused on mothers. This study aimed at: investigating the criterion validity of the Interview of Maternal Representations During Pregnancy-Revised (IRMAG-R) and of the Interview of Paternal Representations During Pregnancy (IRPAG), using EA, parental attachment, and child attachment toward both parents, as criteria; testing the incremental validity of the IRMAG-R and IRPAG in the prediction of child attachment, controlling for other covariates, such as depressive and anxious levels during pregnancy, EA, and parental attachment; evaluating the possible mediation role of EA on the relationship between parental representations during pregnancy and child attachment. Fifty couples of primiparous parents were recruited during pregnancy, when the IRMAG-R and IRPAG were administered to mothers and fathers. At 6–9 months after childbirth, the mother–child and father–child interactions were coded by means of the EA Scales (EAS). At 14–18 after childbirth, the Adult Attachment Interview (AAI) was administered to parents, and the Strange Situation Procedure (SSP) was carried out to assess children's attachment toward mothers and fathers, respectively. The results showed significant correlations between parental pre-natal representations and EA, parental attachment and child attachment. As regards the prediction of child attachment, the IRMAG-R/IRPAG categories showed: a significant and large unique contribution for maternal representations; a close to be significant contribution for paternal representations (with a higher effect size for mothers than fathers). Moreover, while the indirect effect of pre-natal representations in the prediction of child attachment was not significant for mothers, it was instead significant for fathers. The results of this study confirmed the criterion validity of the IRMAG-R and IRPAG, and supported the incremental validity of the IRMAG-R and IRPAG in the prediction of children's attachment categories. Finally, the mediation models revealed that EA did not mediate the relationship between maternal pre-natal representations and child attachment, while it totally mediated the relationship between paternal pre-natal representations and child attachment.

## Introduction

The parent–child relationship begins during pregnancy, when both women and men face dramatic psychological reorganizations related to the new task of becoming parents (1–4). In women, these transformative processes are sustained by the development of mental representations, concerning themselves as mothers, the unborn infant, and the future relationship with him (5–9). Maternal representations become particularly clear and rich by the third trimester of pregnancy (10), when the mothers can fully perceive their infant's vitality thorough intrauterine movements and ultrasound images (1, 5, 11–14). These perceptual experiences allow the mothers to experience a shift from focusing on themselves to their infant as a separate object (15).

Although pre-natal representations have been poorly investigated in men, the existing literature shows that expectant fathers also create an emotional bond with the infant (16–18), and this bond increases as the pregnancy progresses (19–21).

Pre-natal mental representations include projections, dreams, attributions, and fantasies, which are strictly related to parents' childhood attachment experiences (22, 23). During pregnancy, such early experiences become closer to conscious awareness, allowing both women and men to identify with their parents and–at the same time–develop their own parental identity (12, 24). As George and Solomon (25) have underlined, a relevant change takes place in the representational world of expectant parents, whose goals switch from being cared to being caregivers.

A central task for expectant parents is developing a feeling of intimate connection to the infant and, progressively, recognizing him/her as a separate individual who has the need for both care and autonomy (2). Consistent with these considerations, Tambelli et al. (26) have underlined that, when pre-natal representations are flexible and open to change, parents can “have an unconditional acceptance of the infant and a realistic consideration of the baby's individual characteristics and of any difficulties emerging in the relationship with him or her” (pp. 378–379).

Parental pre-natal representations tend to be relatively stable after their formation, serving as a sensitive indicator of the caregiving behaviors (25, 27) and interactive patterns that parents will put in place after childbirth (26, 28, 29).

### Emotional Availability and Child Attachment

After childbirth, the parent–child relationship takes the form of a “reciprocal interchange,” that occurs between the innate propensity of infants to engage with and share the subjective states of others (30, 31) and the ability of parents to interpret and respond appropriately to the emotional underpinnings of their infant's overt behavior (32).

From such a perspective, the theoretical construct of *Emotional Availability* (EA) (33, 34) provides a relevant description of the parent-infant relationship. Such a construct–which is theoretically grounded on attachment theory (35) and integrated with Emde's conceptualization of emotions (36)–refers to the “capacity of a dyad to share an emotional connection and to enjoy a mutually fulfilling and healthy relation” [(37), p. 1]. Scientific literature has documented that EA predicts a wide range of child outcomes, including attachment security [for a review, see (38, 39)].

During the first year of life, repeated interactions with the parents are internalized as an internal working models (IWMs) of attachment (35). IWMs can be regarded as generalized representations of “lived experiences” with primary caregivers (40, 41), that remain fairly stable across the lifespan (42–45).

Early positive emotional interactions allow the children to consider the parents as *secure base* (42), that is as someone who is emotionally available to them in times of distress (35, 46, 47). The internalization of these positive interactive experiences (and of their related affects) promote the development of attachment security in children. In these cases, IWMs include positive expectations about others' EA and willingness to provide support, along with positive representations of the self as competent and valued. Conversely, when parents are not emotionally available and responsive, children develop doubts about their self-worth and others' goodwill, and use defensive strategies other than confident proximity seeking, to face distress. As a result of these negative emotional experiences, two insecure IWMs–*avoidant* or *resistant/ambivalent*–are likely to develop. Avoidance reflects a tendency to use *deactivating* strategies, in response to parents that children perceive as insensitive or rejecting to their attachment needs of reassurance (48). In these cases, children tend to hide or suppress negative emotions (such as anxiety, fear, anger or need for consolation) and deal with distress autonomously to avoid the frustration caused by the potential rejection from the parents (44, 49–52). On the contrary, resistant/ambivalent attachment reflects the use of *hyperactivating* strategies, in response to parents who show inconsistent, hesitant or unpredictable EA toward the emotional needs of their children (48). In these cases, children tend to amplify proximity seeking behaviors to demand or force the parents to be accessible and to pay more attention to them (48, 53).

### Aims of the Study

Whereas, a large body of research has explored the influence of parental post-natal representations on the quality of attachment in children, studies on the predictive role of parents' pre-natal representations on child attachment are still very scarce. Given that pre-natal representations are found to be related to both post-natal representations and post-natal parent–infant interaction (7, 54, 55), it would be important that the influence of parental pre-natal representations on parent–child attachment may also be investigated. At the same time, it is worth noting that, even though research has come to document the relevant influence of fathers on children's development (56, 57), over the past decades, research on parental pre-natal representations have mainly focused on mothers (18). We believe that the lack of studies on fathers represents a further relevant gap within scientific literature, which should be filled by greater attention toward paternal contribution to child socio-emotional development.

Considering the importance of exploring, both in mothers and in fathers, the complex constellations of mental representations during pregnancy as well as their influence on child attachment, this study aimed at:

- investigating the criterion validity of the IRMAG-R and IRPAG, using EA in mother–child and father–child interactions (hereafter referred to as maternal EA and paternal EA), parental attachment, and child attachment toward parents as criteria;
- testing the incremental validity of the IRMAG-R and IRPAG in the prediction of child attachment, with respect to parental depressive and anxious levels during pregnancy, EA scales, and parental attachment;
- evaluating the possible mediation role of EA on the relationship between parental representations during pregnancy and child attachment.

We expected that, both in mothers and in fathers, mental representations during pregnancy will be positively correlated with EA as well as parental and children's attachment categories.

We also expected that, both in mothers and fathers, the categories of pre-natal mental representations will provide a unique incremental contribution in the prediction of children's attachment categories, even when parental depressive and anxious level during pregnancy, EA, and attachment were included as covariates.

Finally, we expected that, both in mothers and in fathers, EA will mediate the effect of pre-natal parental representations on children's attachment.

## Materials and Methods

### Participants

Initially, 189 couples of primiparous parents were recruited at seventh/eighth month of pregnancy, while they were attending childbirth preparation courses at maternity and child health services. These parents had been enrolled in a larger extensive research, aimed at assessing the effects of early interventions on parents at risk for psychopathological symptoms and on their children's socio-emotional development during the first year of life. The screening of parental anxiety and depression revealed 78 couples in which both parents were within the normal range. These parental couples were excluded from the general study and were enrolled in the present investigation. Of these 78 couples, 28 were ruled out because they did not complete all the longitudinal observations. Thus, the final samples consisted of 50 couples of primiparous mothers (mean age = 33.88 years; SD = 4.58) and primiparous fathers (mean age = 36.90 years; SD = 6.69).

The gestation period did not reveal complications for 66% of mothers, and only 8% of them reported that they needed at least one hospitalization. In addition, 20% of mothers reported having had abortions previously. At the time of the study neither mothers nor fathers showed the presence of anxious or depressive symptoms.

This study was carried out in accordance with the recommendations of the Ethics Committee of the Department of Dynamic and Clinical Psychology, “Sapienza” University of Rome. Prior to data collection, the parents received complete information concerning the rationale of the study procedures and provided their written informed consent to participate to the research study, as stated in the Declaration of Helsinki.

### Procedure

The longitudinal study included three measurement occasions, in which different types of instruments were administrated at the Department of Dynamic and Clinical Psychology: semi-structured interviews, self-report scales, and rating scales applied to videotaped materials.

1. At 7–8 month of pregnancy, a sociodemographic interview was administered to the mothers and fathers, with self-reported questionnaires that assessed depressive and anxious symptomatology [i.e., the Edinburgh Post-natal Depression Scale (EPDS) and the State-Trait Anxiety Inventory Y form (STAI-Y), respectively]. Parents also completed semi-structured interviews that assessed their mental representations [i.e., the Interview of Maternal Representations During Pregnancy-Revised (IRMAG-R) and the Interview of Paternal Representations During Pregnancy (IRPAG)].
2. At 6–9 months after childbirth, the mother-child and father-child free-play-home interactions (lasting ~15–20 min) were filmed and coded by means of the Emotional Availability Scale (EAS).
3. At 14–18 months after childbirth, the Adult Attachment Interview (AAI) was administered to both mothers and fathers, and the Strange Situation Procedure (SSP) was carried out to assess the quality of children's attachment toward mothers and fathers, respectively.

### Instruments

*State-Trait Anxiety Inventory Y form* (STAI-Y) (58); Italian version by Pedrabissi and Santinello (59). It is a self-report scale designed to measure both state (Y-1 form) and trait (Y-2 form) anxious subjective states, such as tension, worry, restlessness, nervousness and reactivity. State and trait subscales include 20 items with a four-point Likert scale. The Italian version presented alphas > 0.85 in both adult and adolescent samples. Global scores of state and trait anxious symptoms were computed summing up all 20 items for each scale. The cut-off value for a clinical anxiety level is 40.

*Edinburgh Post-natal Depression Scale* (EPDS) [(60), Italian version by (61)]. Even though this self-report scale was originally developed to measure depressive symptomatology in mothers during the post-natal period, its validity has also been successively demonstrated during pregnancy as well as in its application with fathers. The EPDS includes 10 items that explore the presence of the following depressive symptoms during the past week: inability to laugh, inability to enjoy, unmotivated feelings of guilt, state of anxiety or worry, moments of fear or panic, feeling of being overwhelmed by things, difficulty in sleep due to sadness and unhappiness, feeling of sadness, presence of excessive crying, thinking of getting hurt. The internal consistency of the Italian version of the EPDS was evaluated both with a Cronbach's estimation (alpha = 0.79) and Guttman split-half index (r_tt_ = 0.82). A Global Score for depressive symptomatology was computed summing up all items. The cut-off value for the Italian version of the scale is 12/13 for clinical depression and 9/10 for screening purposes.

*Interview of Maternal Representations During Pregnancy-Revised* (IRMAG-R) (8, 9, 62) and *Interview of Paternal Representations During Pregnancy* (IRPAG) (28). These semi-structured interviews consist of 47 open questions, designed to assess maternal and paternal representations during the third trimester of pregnancy, by examining parental narratives regarding the future child and the unfolding of the relationship with him/her. Parental narratives are coded as a function of seven different dimensions (i.e., richness of perceptions, openness to change, intensity of investment, coherence, differentiation, social dependency, and dominance of fantasies), that allow the mothers' and fathers' transcripts to be classified into one of three categories: *Integrated/Balanced, Restricted/Disengaged* and *Not Integrated/Ambivalent*. The *Integrated/Balanced* category is characterized by the ability of parents to provide a consistent picture of their experience in the context of their personal history; they give rich, affectively involved and flexible representations of their children, even though still unborn, and of their future with him/her. Parents consider pregnancy as an important step of personal development and the fulfillment of their personal identity. *Restricted/Disengaged* category is characterized by rigid representations, impersonality, poor fantasies, and high emotional control and inhibition. Moreover, restricted/disengaged parents usually show difficulty imagining and managing the relationship with their children, and recognizing the experience of pregnancy. Finally, in *Not Integrated/Ambivalent* category, parents tend to report not organized and poorly coherent narratives, in which different tendencies toward parenthood and the child coexist (defined by excessive involvement and the struggle to impose distances), as they are strongly absorbed by their conflicts with their original family or partners. The degree of inter-rater reliability for all dimensions as estimated in terms of agreement between judges was: 0.86 for Richness of Perceptions; 0.89 for Openness to Change; 0.90 for Intensity of Investment; 0.84 for Coherence; 0.93 for Differentiation; 0.97 for Social Dependency, and 0.86 for Dominance of Fantasies, confirming the high level of reliability of the instrument (62).

*Emotional Availability Scales* (EAS) (34). The EAS coding system [EAS 4th Edn; (63)] was applied to 15/20 min of video-recorded free-play home-interactions. The instrument was composed of six scales designed to assess different dimensions of parent–child emotional regulation. Four scales concern parental EA toward children (*Sensitivity, Structuring, Non-Intrusiveness*, and *Non-Hostility*), and two concern children's EA toward parents (*Responsiveness* and *Involvement*), with a range from one (highly emotional unavailable) to seven (highly emotional available) points. *Sensitivity* refers to parental affectivity, acceptance, flexibility, clarity of perceptions, affect regulation, and variety and creativity that was shown during play toward children. *Structuring* refers to parental capacity to give rules, regulations and a supportive framework for interaction. *Non-Intrusiveness* refers to parental capacity to interact with the child without being over-directive, over-stimulating or overprotective. *Non-Hostility* concerns covert and overt parental hostility. *Responsiveness* refers to children's availability toward their parents' requests of interaction, along with children's enjoyment of the interaction. *Involvement* regards children's willingness to interact with their parents. Inter-rater reliability, assessed with mean absolute agreement intraclass correlation coefficients (ICC), ranged from 0.81 to 0.93.

*Adult Attachment Interview* (AAI) (64). The AAI is a semi-structured interview formed by 20 questions requesting respondents to describe their relationship with main attachment figures during childhood, specific positive or negative memories, traumas, and current attachment relationships. Some questions specifically concern crucial events related to attachment relationships, such as illnesses, separations and rejections. Adult participants are asked to recall autobiographical memories from early childhood in order to evaluate the narratives produced, by considering the structural dimension of the transcript rather than its content. The AAI coding system was applied to categorize participants into one of five categories corresponding to different states of mind with respect to attachment: *Secure/Autonomous (F)*; *Dismissing (Ds)*; *Preoccupied (E)*; *Unresolved/Disorganized (U)*; *Cannot Classify (CC)*. The *F* classification includes individuals who value attachment relationships, describe their attachment experiences (whether positive or negative) coherently and consider them important for their own personality. In the *DS* classification, adults tend to minimize the importance of attachment for their own lives or to idealize their childhood experiences. Adults classified as *E* tend to maximize the importance of attachment, are still very much involved and preoccupied with their past experiences, and are unable to describe them coherently and reflectively. Anger or passivity characterizes the discourse style of these adults. The additional classification *U* is applied to interviewees who show signs of unresolved experiences of trauma usually involving the loss of attachment figures. Finally, the *CC* classification is applied when a transcript has strong characteristics of both the dismissing and preoccupied categories. Inter-rater reliability with respect to the main category was 89% with a *k* = 0.74, *p* = 0.001.

*Strange Situation Procedure* (SSP) (46). The SSP is a standardized laboratory observational procedure, commonly carried out between 12 and 18 months after childbirth, during which the child's attachment behavior toward his/her parent is activated and intensified by the child's exposure to a moderately, yet increasingly stressful situation (i.e., the presence of a strange person and two short separations from the mother). The SSP originally classified infants into three categories: *Secure (B)*; *Insecure Avoidant (A)*; and *Insecure Resistant/Ambivalent Attachment (C)*. The *B* classification characterizes children who use parents as a secure basis when they are present, show distress when were separated from them and actively seek contact when they return with a certain predisposition to be easily consoled. The *A* classification characterizes children who do not seek contact and play with parents, and do not show distress when are separated from them. During the reunion with the parents, these children are not interested in seeking proximity to them, manifest a tendency to avoid contact with them and continue to play or to explore the environment. Moreover, they are not disturbed in the presence of the unfamiliar adult (the stranger) and during the entire procedure. Finally, The *C* classification characterizes children who are strongly focused on parents during SSP, show reluctance to explore the environment, and express high levels of distress during the separations from the parents as well as inconsolability during the reunions with them. Main and Solomon (53) later added a fourth category, *Disorganized/Disoriented (D)* defined by odd, awkward behavior and unusual fluctuations between anxiety and avoidance. As reported by George and Solomon (65), when coders are trained to categorize attachment styles using all categories the percentage of agreement between judges was from 80 to 88%.

### Data Analysis

To investigate the criterion validity of the IRMAG-R and IRPAG categorizations, point-biserial correlations (for relationships between dichotomous and scale variables) and phi correlations (for relationships between dichotomous variables) were used.

To investigate the incremental validity of the IRMAG-R and IRPAG categorizations in the prediction of children's attachment, with respect to levels of anxiety and depression during pregnancy, parent–child EA, and adult attachment), two logistic regressions were conducted.

Finally, to investigate the mediation role of parent–child EA on the relationship between parental representations during pregnancy and children's attachment patterns, two mediation models were analyzed (one for mothers and one for fathers) through Mplus software, Version 8 (66), using the weighted least square mean and variance adjusted estimator (WLSMV), which allows the computation of the indirect effects also with dichotomous outcomes. Moreover, a biased corrected estimation of confidence intervals of parameters through a bootstrap procedure was used.

As regards statistical power, it is worth noting that medium/large effect sizes are expected for the relationships among the main factors investigated (i.e., parental representations during pregnancy, EA and children's attachment patterns). In this view, to reach a statistical power of 0.80 with medium/large effects and a level of alpha to 0.05, the number of subjects needed is about 50 subjects for both logistic regressions [G^*^Power software; (67)] and mediation models with biased corrected confidence intervals for indirect effects' parameters (68).

## Results

### Descriptive Statistics

Table 1 illustrates the frequency and percentages for each category of the IRMAG-R and IRPAG, maternal AAI and paternal AAI, and SSP, as carried out with the mothers and fathers, respectively (hereafter referred as maternal SSP and paternal SSP).

**Table 1:** Frequency and percentages of IRMAG-R and IRPAG, maternal and paternal AAI, and maternal and paternal SSP categories.

	**Integrated/balanced**	**Restricted/disengaged**	**Not integrated/ambivalent**
IRMAG-R	26 (52%)	11 (22%)	13 (26%)
IRPAG	26 (52%)	15 (30%)	9 (18%)
	**Secure/autonomous**	**Dismissing**	**Preoccupied**
Maternal AAI	29 (58%)	15 (30%)	6 (12%)
Paternal AAI	27 (54%)	16 (32%)	7 (14%)
	**Secure**	**Avoidant**	**Ambivalent**
Maternal SSP	34 (68%)	8 (16%)	8 (16%)
Paternal SSP	27 (54%)	16 (32%)	7 (14%)

*IRMAG-R, Interview of Maternal Representations During Pregnancy-Revised; IRPAG, Interview of Paternal Representations During Pregnancy; Maternal AAI, Maternal Adult Attachment Interview; Paternal AAI, Paternal Adult Attachment Interview; Maternal SSP, Strange Situation Procedure carried out with the mothers; Paternal SSP, Strange Situation Procedure carried out with the fathers*.

As regards the narratives about parenthood during pregnancy, more than 50% of representations were Integrated/Balanced for both mothers and fathers. Restricted/Disengaged representations were slightly higher for fathers, while Not Integrated/Ambivalent were slightly higher for mothers.

Regarding parental AAI, more than 50% of both mothers and fathers showed a Secure attachment. The number for Dismissing attachment was higher than that for Preoccupied for both mothers and fathers.

As regards the SSP procedure, more than 50% of the children showed a Secure attachment both toward mothers and fathers. However, a higher percentage of Secure attachment emerged toward mothers rather than toward fathers. The number of children's Insecure Avoidant attachment was two times higher toward fathers rather than toward mothers, while a similar number for Insecure Resistant/Ambivalent attachment was found toward mothers and fathers.

Table 2 shows the descriptive statistics of all scales considered in the present study, with mean scores, standard deviation as well as skewness and kurtosis values for both maternal and paternal scales. Interestingly, mean values are higher for maternal than paternal scores in all scales, especially for trait anxiety (STAI-TRAIT) and depressive symptoms. All scales showed close to normal distribution, except for the kurtosis value of maternal STAI-STATE and scores on EAS related to father–child interactions, which revealed values higher than one.

**Table 2:** Descriptive statistics of quantitative scales.

	**Maternal mean**	**Paternal mean**	**Maternal SD**	**Paternal SD**
STAI-STATE	34.88	32.5	8.75	7.03
STAI-TRAIT	37.54	32.82	7.72	7.89
EPDS	6.34	3.58	4.02	2.94
EAS	5.78	5.57	0.84	0.91

*STAI-STATE, State-Trait Anxiety Inventory State Form; STAI-TRAIT, State-Trait Anxiety Inventory Trait Form; EPDS, Maternal Edinburgh Post-natal Depression Scale; EAS, Emotional Availability Scale*.

### Criterion Validity of the IRMAG-R and IRPAG

To evaluate the criterion validity of the IRMAG-R and IRPAG, the correlations of maternal and paternal representations during pregnancy with parental EA, and with parental and children's attachment were estimated.

Since some of the IRMAG-R, IRPAG, AAI and SSP categories showed a too low frequency to conduct appropriate statistical analysis (e.g., see Table 1 referring to Not Integrated/Ambivalent, Preoccupied and Resistant/Ambivalent categories), Restricted/Disengaged, Dismissing and Avoidant categories were collapsed, respectively with Not Integrated/Ambivalent, Preoccupied and Resistant/Ambivalent ones. In this way, parental representations were divided into Integrated vs. Not Integrated categories, parental attachment models into Secure vs. Insecure categories and children's attachment patterns into Secure vs. Insecure categories.

As illustrated in Table 3, the IRMAG-R and IRPAG categories (Integrated vs. Not Integrated representations) were positively and significantly correlated (phi correlation) with maternal and paternal AAI and SSP categories, with a high effect size, providing support for their criterion validity. In Table 3 point-biserial correlations between the IRMAG-R/IRPAG categories and EAS scores are also reported. Significant and positive correlations were found between parental representations during pregnancy and parental EAS with a high effect size, with a further support for the criterion validity of IRMAG/IRPAG interviews.

**Table 3:** Correlations of IRMAG-R and IRPAG with criteria.

	**Maternal/Paternal EAS**	**Maternal/Paternal AAI**	**Maternal/Paternal SSP**
IRMAG-R	0.676**	0.237	0.628**
IRPAG	0.540**	0.318*	0.479**

IRMAG-R, Interview of Maternal Representations During Pregnancy-Revised; Maternal EAS, Maternal Emotional Availability Scale; Maternal AAI, Maternal Adult Attachment Interview. IRPAG, Interview of Paternal Representations During Pregnancy; Paternal EAS, Paternal Emotional Availability Scale; Paternal AAI, Paternal Adult Attachment Interview. ^*^significant effect at 0.05 alpha level (two tails); ^**^significant effect at 0.01 alpha level (two tails).

### Incremental Validity of the IRMAG-R and IRPAG in the Prediction of Child Attachment

In order to investigate the incremental validity of the IRMAG-R and IRPAG, two logistic regressions (one for mothers and one for fathers) were conducted, including parental representations during pregnancy (Integrated vs. Not Integrated), EA and the parental attachment model (Secure vs. Insecure) as predictors, children's attachment (Secure vs. Insecure) as a criterion, and age and children's gender as covariates. Parental anxious and depressive scores were excluded from these analyses, as they did not show significant correlations neither with IRMAG-R/IRPAG, nor with parental and children's attachment and with EA scales.

As illustrated in Table 4, overall maternal predictors accounted for a considerable portion (*R*^2^ Nagelkerke = 0.54) of maternal SSP variability. Moreover, the Hosmer and Lemeshow test indicated an adequate fit for the model [$\chi_{\left(8\right)}^{2}$ = 5.09, *p* = 0.75].

**Table 4:** Logistic regression on maternal SSP attachment.

	**B**	**SE**	**Wald (1 df)**	**p**	**OR**	***R*^**2**^ Nagelkerke**
Mothers' age	0.07	0.09	0.60	0.44	1.07	
Children's gender	0.63	0.87	0.52	0.47	1.88	
IRMAG-R	3.76	1.31	8.18	0.00	42.73	
Maternal EAS	0.04	0.62	0.01	0.95	1.04	0.54
Maternal AAI	0.06	0.87	0.00	0.95	1.06	
Intercept	−3.53	4.62	0.58	0.45	0.03	

*IRMAG-R, Interview of Maternal Representations During Pregnancy-Revised; Maternal EAS, Maternal Emotional Availability Scale; Maternal AAI, Maternal Adult Attachment Interview*.

As regards the single predictors, a significant unique contribution emerged for the IRMAG-R with a high effect size in terms of Odds Ratio, while not significant unique contributions were found for maternal AAI, EAS, as well as for children's gender and maternal age.

Similarly, paternal predictors accounted for a high portion (*R*^2^ Nagelkerke = 0.64) of paternal SSP variability (See Table 5), and the Hosmer and Lemeshow test again indicated an adequate fit for the model [$\chi_{\left(8\right)}^{2}$ = 8.48, *p* = 0.39]. As regards single predictor effects, a significant unique contribution emerged for paternal EAS, with a high effect size (as evaluated in terms of Odds Ratio), as well as a close to be significant contribution (with a large Odds Ratio) of the IRPAG categories. Not significant contributions were found for AAI categories, children's gender and paternal age (Table 5).

**Table 5:** Logistic regression on paternal SSP attachment.

	**B**	**SE**	**Wald**	**p**	**OR**	***R*^**2**^ Nagelkerke**
Paternal age	−0.03	0.07	0.18	0.67	0.97	
Children's gender	0.60	0.89	0.45	0.50	1.82	
IRPAG	1.62	1.04	2.44	0.12	5.07	
Paternal EAS	3.45	1.13	9.24	0.00	31.36	0.64
Paternal AAI	−2.07	1.17	3.10	0.08	0.13	
Intercept	−18.09	6.50	7.76	0.01	0.00	

*IRPAG, Interview of Paternal Representations During Pregnancy; Paternal EAS, Paternal Emotional Availability Scale; Paternal AAI, Paternal Adult Attachment Interview*.

### Direct and Indirect Effects of Parental Representations During Pregnancy on Child Attachment: the Mediating Role of EA

In order to further investigate the effect of parental representations during pregnancy on children's attachment, two mediation models were tested (one for mothers and one for fathers), including the IRMAG-R/IRPAG categories (Integrated vs. Not Integrated) as independent variables, maternal and paternal EAS scores as mediators, SSP categories (Secure vs. Insecure) toward mothers and fathers as dependent variables, and maternal and paternal AAI categories (Secure vs. Insecure) as covariates. These mediation analyses were conducted with Mplus Version 8 software using the WLSMV estimator and bootstrapping procedure to estimate confidence interval of indirect effects.

**Figure 1:**
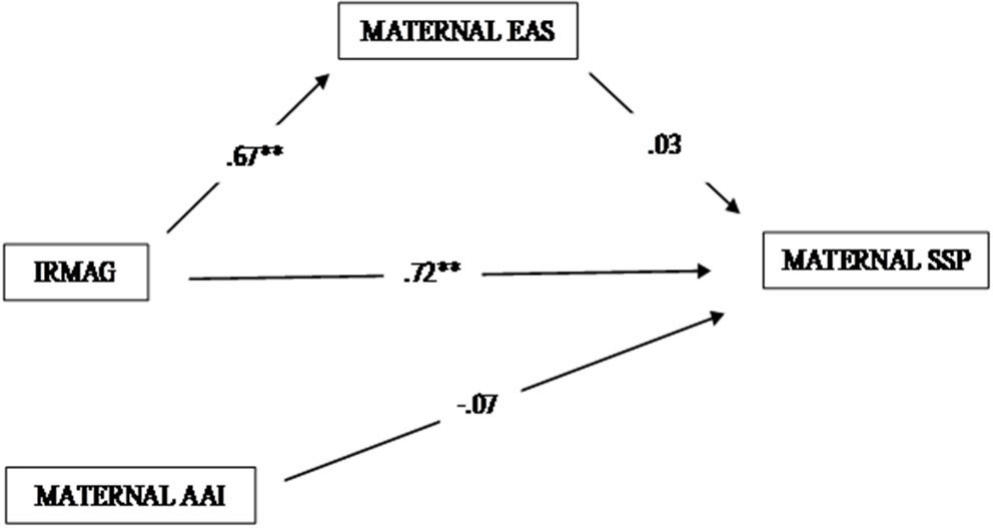
Mediation model on children's attachment toward mothers. **significant effect at 0.01 alpha level (two tails).

The first mediation model, which included maternal variables, showed an adequate fit with the data [$\chi_{\left(1\right)}^{2}$ = 0.17, *p* = 0.68; RMSEA = 0.00 (0.00–0.28); CFI = 1.00; TLI = 1.12; WRMR = 0.14]. The model accounted for 53% of children's attachment variability, and for 46% of maternal EA. As illustrated in Figure 1, results showed significant direct effects of IRMAG-R categorizations on both EAS scores (Biased Corrected Bootstrap 99% CI: from 0.47 to 0.82) and SSP categories (Biased Corrected Bootstrap 99% CI: from 0.07 to 1.10), and not significant effects of EAS scores (Biased Corrected Bootstrap 99% CI: from −0.37 to 0.62) and maternal AAI categories on SSP ones (Biased Corrected Bootstrap 99% CI: from −0.61 to 0.24).

Moreover, a not significant indirect effect (Estimate = 0.02, Estimate/SE = 0.18, *p* < 0.86) of the IRMAG-R categorizations, via maternal EAS, on children's attachment toward mothers was found (Biased Corrected Bootstrap 99% CI: from −0.25 to 0.45), indicating that the impact of maternal representations during pregnancy on children's attachment was exclusively direct.

**Figure 2:**
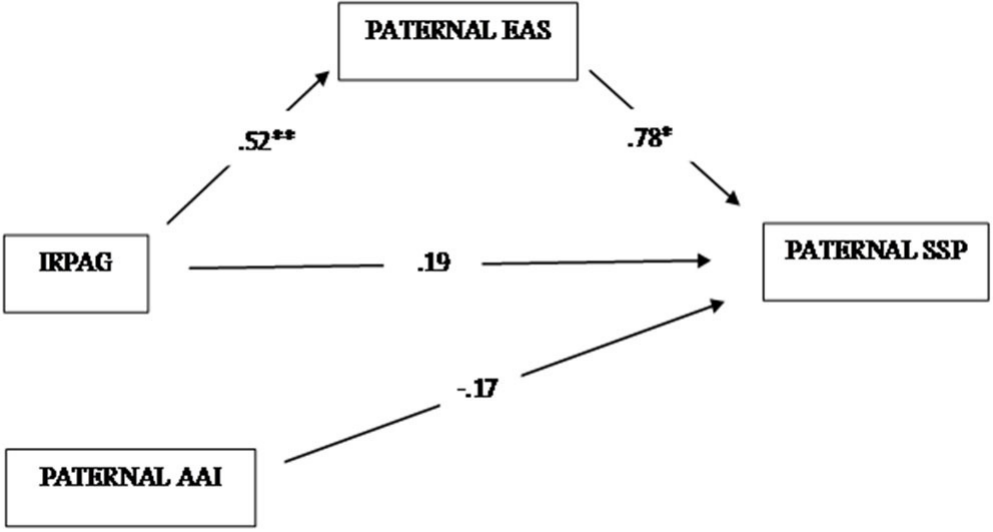
Mediation model on children's attachment toward fathers. *significant effect at 0.05 alpha level (two tails); ** significant effect at 0.01 alpha level (two tails).

Similar to the first model, the second one, which included paternal variables, also showed an adequate fit with the data [$\chi_{\left(1\right)}^{2}$ = 0.55, *p* = 0.46; RMSEA = 0.00 (0.00–0.34); CFI = 1.00; TLI = 1.05; WRMR = 0.26]. The model accounted for 76% of children's attachment variability, and for 28% of paternal EA. As illustrated in Figure 2, a significant direct effect of the IRPAG categories on EAS scores was found (Biased Corrected Bootstrap 99% CI: from 0.22 to 0.71), along with a significant effect of the EAS scores on children's attachment (Biased Corrected Bootstrap 99% CI: from 0.47 to 1.03). Conversely, not significant direct effects of both IRPAG (Biased Corrected Bootstrap 99% CI: from −0.22 to 0.55) and paternal AAI categories on children' attachment (Biased Corrected Bootstrap 99% CI: from −0.57 to 0.24) were found.

Different from the first model, a significant indirect effect (Estimate = 0.41, Estimate/SE = 4.39, *p* < 0.001) of the IRPAG categories, via paternal EA, on children's attachment toward fathers was found (Biased Corrected Bootstrap 99% CI: from 0.19 to 0.68), indicating that the impact of paternal representations during pregnancy on children's attachment is totally indirect.

## Discussion

Whereas the influence of parental post-natal representations on child attachment has been extensively investigated, much less is known about the predictive role of parental pre-natal representations on child attachment. Moreover, the existing literature on pre-natal mental representations have been mainly focused on mothers (18), while paternal mental representations during pregnancy have received scarce attention from the research.

Beginning from these premises, we firstly investigated the criterion validity of the IRMAG-R and IRPAG, using maternal EA, paternal EA, parental attachment, and child attachment toward parents as criteria.

Consistent with our expectations, both in mothers and in fathers, the results showed a strong relationship between pre-natal representations and EA, and between pre-natal representations and child attachment categories (Table 3). These associations are supported by the evidence that parental pre-natal expectations, thoughts, and fantasies shape an *anticipatory working model* (6, 69), that sustains women and men in the achievement of a parental identity and in the development of an early attachment bond with their infants (70). Consistent with the results of previous investigations (27, 71, 72), small size correlations (in terms of Cohen's standards) were also found between pre-natal representations and parental attachment, with a further support for the criterion validity of the IRMAG-R and IRPAG. These results may be interpreted taking into account the role played by the reworking of early attachment relationship in enabling expectant mothers and fathers to achieve their own parental identity and develop the capacity to recognize the unborn infant as a separate individual with specific needs (2). Parents with positive and stable childhood experiences are more likely to develop and maintain flexible and coherent representations about attachment and caregiving during the transition to parenthood (1).

Having explored the criterion validity of parental pre-natal representations, we tested the incremental validity of the IRMAG-R and IRPAG in the prediction of child attachment, with respect to children's gender, parental age, EA scales, and parental attachment.

Contrary to our expectations, even though maternal and paternal predictors accounted for a considerable portion of variability of attachment categories in children, relevant differences between mothers and fathers were found regarding the effects of the single predictors. As regards the mothers, the categories of mental representation during pregnancy provided a significant unique incremental contribution in the prediction of children's attachment categories, while all the other considered predictors did not provide a unique contribution (Table 4). These results evidenced that, compared to children whose mothers have not integrated pre-natal representations, children whose mothers report integrated representations during pregnancy have a higher possibility to develop a sense of security in the attachment relationship. Thus, in our study, maternal pre-natal representations have a specific role in the construction of the attachment bond with the child. Different from that observed in mothers, in the case of fathers, child attachment categories were accounted for by the significant unique contribution of EA during dyadic interactive exchanges. Moreover, fathers' pre-natal representations resulted in providing a weaker (close to be significant) unique contribution to child attachment if compared to maternal representations (Table 5). These results show that children's attachment security toward fathers is more likely to be associated with high EA during dyadic interactive exchanges. Undoubtedly, the results concerning fathers confirmed those of previous investigations that have documented the role of parent–child EA in predicting secure attachment in children (38, 39). At the same time, it is worth noting that, even though pre-natal representations resulted in predicting child attachment both in mothers and (with a lower effect size) in fathers, only in mothers the other predictors did not provide any unique contribution.

Finally, taking into account the peculiarities between maternal and paternal contributions on child attachment, we evaluated the possible mediation role of EA on the relationship between parental representations during pregnancy and child attachment.

Even in this case, the analyses produced unexpected findings. As regards the mothers, results showed significant direct contributions of mental representations in predicting both EA and child attachment categorizations, while no direct effects were found for EA and maternal attachment on child attachment categories. Moreover, no indirect effect of maternal pre-natal representations, via EA, on children's attachment categories was found (Figure 1). Different from that observed in mothers, in fathers, a significant direct effect of pre-natal representations on EA was found, along with a significant effect of EA on children's attachment category. Conversely, neither paternal pre-natal representations nor paternal attachment category resulted in having a direct effect on children's attachment categories (Figure 2).

The results of our study may be explained by taking into account the well-known condition of *primary maternal preoccupation* (73), that has been conceptualized as “almost an illness” that a mother must experience and recover from, in order to provide the infant with an environment that can meet his/her physical and psychological needs. As Leckman et al. (74) have evidenced, such preoccupations develop during the last months of pregnancy, affecting both mothers and (to a lesser extent) fathers, with the aim of heightening parental ability to anticipate the infant's needs, learn his/her emotional signals, and gradually recognize him/her as an individual. It may be assumed that, because of more intense preoccupations, mothers may be more prone than fathers to develop vivid mental representations of their infants and an early sensitive attitude toward them (75). The results of our study seem to prove that these maternal inclinations are so consolidated during pregnancy as to shape the ground in which the child's sense of security will be rooted.

In mothers, the experience of a *somatic gestation* (26) contributes consistently in increasing the richness and specificity of mental representations about their unborn infants. During pregnancy, maternal mental representations are sustained by the perception of the baby, whose vitality is manifested through intrauterine movements and ultrasound images (1, 5, 11–14). The father's emotional relationship with the unborn infant is instead *indirect*, as it is experienced via the mother's willingness to share with them the affective and somatic experience of pregnancy (76). In this view, it may be assumed that fathers' contribution to their child's attachment security may fully emerge only when they will have the possibility to *really* interact with their *real* child (77).

### Limitations and Strengths of the Study

The main constraint of this study is the small number of the recruited parents, as it produced an increase of parameters' standard errors and a decrease of statistical power that limited the possibility to detect low size effects. The small number of participants did not even permit to test the effect of some potentially relevant variables, such as previous abortions (which was reported only by 10 mothers).

As regards the mediation analysis, it is worth noting that all variables included in the model were assessed only on one occasion of measurement. As a consequence, the analyzed models did not include residual change estimations of both mediator and outcome (as computed using autoregression-based statistical procedures), with a possible distortion of parameter estimations (for an extensive explanation, see (78). Further longitudinal studies with multiple measurements of all variables are needed to address this potential source of distortion.

In this study, we adopted a dyadic perspective to evaluate (separately for mothers and fathers) the predictive role played by pre-natal representations on child attachment. This did not allow examination whether pre-natal triadic family relations might predict mother- and father-child attachment relationship. As regard this issue, a recent investigation has shown that children's attachment toward fathers (but not toward mothers) is predicted by pre-natal triadic family alliance, that is by the ability of the mother and father to cooperate and support each other in their parental roles (79).

Notwithstanding these limitations, no previous study has ever investigated the predictive and incremental validity of maternal and paternal pre-natal representations on child attachment. We believe that our results (albeit preliminary) may provide the starting point for future researches, aimed at shedding further light on the distinct (even though complementary) paths, that mothers and fathers follow to contribute to their children' attachment security.

These reflections lead us to consider the inclusion of fathers (who have been long overlooked in scientific literature on parenting) as a further strength of our study.

Finally, we believe that, in this study, the combined use of clinical semi-structured interviews and observational procedures may have provided an articulated description of the complexity underlying the construction of mother- and father-child attachment relationship.

## Conclusions

The results of this study may have relevant implications for prevention, clinical practice, and future researches, as they indicate pregnancy as a privileged time for the intervention programs that may be designed to support the parents in creating that *intersubjective matrix* (80), within which the child's sense of security develops.

The assessment of mental representations during pregnancy provides the opportunity to recognize parents who will have non-optimal interactions with their infants, after childbirth. Indeed, the IRMAG-R and IRPAG, beyond assessing the emotional valence of parental representations, also allow to identify the presence of defensive strategies (toward pregnancy and the unborn infant) that are sensitive predictors of early impairments in parental EA. These aspects are particularly evident among parents with psychopathological symptoms (26) and with whom this study needs to be replicated.

## Data Availability Statement

The datasets generated for this study are available on request to the corresponding author.

## Ethics Statement

Prior to data collection, the participants received complete information concerning the rationale of the study procedures and provided their written informed consent to participate to the research study, as stated in the Declaration of Helsinki.

## Author Contributions

RT: conceived the work, monitored data acquisition, and provided a substantial contribution to the interpretation of the data. As first author, she was primarily accountable for all aspects of the work. CT: wrote the Introductions, Discussion, and Conclusions sections, revised the paper for intellectual content, and approved its final version to be published. FD: analyzed data, wrote the Methods and Results sections, revised the paper for intellectual content, and approved its final version to be published. All authors agreed to be accountable for all aspects of the work and to ensure that questions related to the accuracy or integrity of any part of the work were appropriately investigated and resolved.

## Conflict of Interest

The authors declare that the research was conducted in the absence of any commercial or financial relationships that could be construed as a potential conflict of interest.
